# *Centella asiatica* Alters Metabolic Pathways Associated With Alzheimer’s Disease in the 5xFAD Mouse Model of *ß*-Amyloid Accumulation

**DOI:** 10.3389/fphar.2021.788312

**Published:** 2021-12-16

**Authors:** Alex B. Speers, Manuel García-Jaramillo, Alicia Feryn, Donald G. Matthews, Talia Lichtenberg, Maya Caruso, Kirsten M. Wright, Joseph F. Quinn, Jan F. Stevens, Claudia S. Maier, Amala Soumyanath, Nora E. Gray

**Affiliations:** ^1^ Department of Neurology, Oregon Health & Science University, Portland, OR, United States; ^2^ Department of Environmental and Molecular Toxicology, Oregon State University, Corvallis, OR, United States; ^3^ Linus Pauling Institute, Oregon State University, Corvallis, OR, United States; ^4^ Department of Chemistry, Oregon State University, Corvallis, OR, United States; ^5^ OHSU-PSU School of Public Health, Oregon Health & Science University, Portland, OR, United States; ^6^ Parkinson’s Disease Research Education and Clinical Care Center, Veterans’ Administration Portland Health Care System, Portland, OR, United States; ^7^ Department of Pharmaceutical Sciences, Oregon State University, Corvallis, OR, United States

**Keywords:** *Centella asiatica*, Alzheimer’s disease, 5xFAD, metabolomics, metabolic pathways

## Abstract

*Centella asiatica* is an herb used in Ayurvedic and traditional Chinese medicine for its beneficial effects on brain health and cognition. Our group has previously shown that a water extract of *Centella asiatica* (CAW) elicits cognitive-enhancing effects in animal models of aging and Alzheimer’s disease, including a dose-related effect of CAW on memory in the 5xFAD mouse model of *ß*-amyloid accumulation. Here, we endeavor to elucidate the mechanisms underlying the effects of CAW in the brain by conducting a metabolomic analysis of cortical tissue from 5xFAD mice treated with increasing concentrations of CAW. Tissue was collected from 8-month-old male and female 5xFAD mice and their wild-type littermates treated with CAW (0, 200, 500, or 1,000 mg/kg/d) dissolved in their drinking water for 5 weeks. High-performance liquid chromatography coupled to high-resolution mass spectrometry analysis was performed and relative levels of 120 annotated metabolites were assessed in the treatment groups. Metabolomic analysis revealed sex differences in the effect of the 5xFAD genotype on metabolite levels compared to wild-type mice, and variations in the metabolomic response to CAW depending on sex, genotype, and CAW dose. In at least three of the four treated groups (5xFAD or wild-type, male or female), CAW (500 mg/kg/d) significantly altered metabolic pathways related to purine metabolism, nicotinate and nicotinamide metabolism, and glycerophospholipid metabolism. The results are in line with some of our previous findings regarding specific mechanisms of action of CAW (e.g., improving mitochondrial function, reducing oxidative stress, and increasing synaptic density). Furthermore, these findings provide new information about additional, potential mechanisms for the cognitive-enhancing effect of CAW, including upregulation of nicotinamide adenine dinucleotide in the brain and modulation of brain-derived neurotrophic factor. These metabolic pathways have been implicated in the pathophysiology of Alzheimer’s disease, highlighting the therapeutic potential of CAW in this neurodegenerative disease.

## 1 Introduction

Alzheimer’s disease (AD) is the most common cause of dementia in the elderly population and is the sixth leading cause of death in the United States (NIoA NIoH, 2020). The financial burden of AD on US health care systems was estimated to be $305 billion in 2020, with an expectation that this number will increase to more than $1 trillion by 2050 as the US population ages (Wong, 2020). Due to the highly complex pathology of AD, development of effective treatments has been difficult (NIoA NIoH, 2020). Prior to the Food and Drug Administration’s recent approval of aducanumab, the first beta-amyloid (Aβ) targeted therapy for AD (Yang and Sun, 2021), only five medications had been approved for AD, all of which offer only symptomatic relief and do not influence disease progression (Alzheimer’s Association, 2020).

The lack of effective treatments is in part due to the multifaceted nature of AD pathophysiology. In addition to characteristic Aβ plaques and tau pathology, recent research suggests that disruptions in other fundamental biological processes, including neuroinflammation (Heneka et al., 2015) as well as mitochondrial and antioxidant pathways (Jiang et al., 2016; Wojsiat et al., 2018; Wang et al., 2020), also play key roles in the progression of the disease. Additionally, there is significant crosstalk between each of these pathways. This has led to a growing interest in identifying therapeutic interventions with multiple biological targets for use in AD.

Herbal therapies offer promising therapeutic potential in AD as natural sources of a diverse array of phytochemical components and targets of action. The medicinal plant *Centella asiatica* (L.) Urb. [Apiaceae], also known as gotu kola, is one such example with a long history of use in traditional Chinese and Ayurvedic medicine for its purported effects on brain health (Gray et al., 2017a). Preclinical and clinical evidence widely supports the cognitive-enhancing and neuroprotective effects of *Centella asiatica*, though the mechanisms underlying these effects are still in question (Gray et al., 2017a). Our group has demonstrated the cognitive-enhancing effects of a water extract of *Centella asiatica* (CAW) in mouse models of aging and AD. (Soumyanath et al., 2012; Gray et al., 2015; Gray et al., 2016; Gray et al., 2018a; Gray et al., 2018b; Matthews et al., 2019; Zweig et al., 2021) These effects were associated with increased antioxidant response and improved mitochondrial function in the brain (Gray et al., 2015; Gray et al., 2016; Gray et al., 2018a; Gray et al., 2018b; Matthews et al., 2019; Zweig et al., 2021).

In a previous study, we examined the effects of increasing concentrations of CAW in the 5xFAD mouse model of Aβ accumulation (Matthews et al., 2019). CAW (0, 200, 500, or 1,000 mg/kg/d) was administered in the drinking water to eight-month-old male and female 5xFAD mice and their wild-type (WT) littermates for 5 weeks. A dose-dependent improvement in memory was observed following CAW treatment for both sexes and genotypes. Memory improvements were associated with significant changes in antioxidant gene expression in both 5xFAD and WT mice, in the absence of a significant effect of CAW on Aβ plaque burden in the 5xFAD mice.

The present study seeks to expand on the previous study in 5xFAD and WT mice by analyzing alterations in the brain metabolome of those same animals to identify possible novel mechanisms of action underlying the cognitive-enhancing effects of CAW. Using high-performance liquid chromatography-high resolution tandem mass spectrometry (HPLC-HRMS/MS), we analyzed the metabolomic profile of cortical tissue collected from the female and male 5xFAD mice and WT littermates and investigated changes in 120 identified metabolites and their related metabolic pathways.

## 2 Materials and Methods

### 2.1 Production and Administration of CAW

CAW was prepared from the dried aerial parts of *Centella asiatica* herb (Lot # 170300206; Oregon’s Wild Harvest, Redmond, OR) that was authenticated as described previously (Matthews et al., 2019). A voucher sample of the original plant material is deposited at the Oregon State University Herbarium (OSC-V-258629). Several batches of a crude water extract (CAW *a*-θ) were prepared as needed from the same lot of plant material using a standardized extraction method described previously (Matthews et al., 2019; Matthews et al., 2020); A voucher sample of each batch is stored at −20°C in our laboratory. Targeted and untargeted HPLC-HRMS/MS (Alcazar Magana et al., 2020) was performed on a representative CAW batch (CAW-iota). Results of targeted analysis of caffeoylquinic acids and triterpenes in CAW iota are shown in Table 1, while untargeted HPLC-HRMS/MS data has been archived at Oregon State University. Animals were provided with CAW (0, 2, 5, or 10 g/L) ad libitum in their water bottles for 5 weeks. We calculated dosing ranges based on the average daily decrease in water volume and denoted these treatments as 0, 200, 500, or 1,000 mg of CAW per kg of body weight per day.

**TABLE 1:** HPLC-HRMS/MS quantification of phytochemicals in a representative batch of *Centella asiatica* water extract (CAW).

Compound	% w/w
*Caffeoylquinic acids*	
1,3-dicaffeoylquinic acid	0.067
1,5-dicaffeoylquinic acid	0.064
Chlorogenic acid	0.525
Isochlorogenic acid A	0.229
Isochlorogenic acid B	0.360
Isochlorogenic acid C	0.264
Neochlorogenic acid	0.149
Total caffeoylquinic acids	1.657
*Triterpenes*	
Asiatic acid	0.057
Asiaticoside	2.387
Madecassic acid	0.094
Madecassoside	1.864
Total triterpenes	4.401

% w/w (percent weight per weight).

### 2.2 Animals

All animal procedures were conducted in accordance with the NIH Guidelines for the Care and Use of Laboratory Animals and were approved by the Institutional Animal Care and Use Committee of the Portland VA Medical Center (IACUC #: 3260-17). 5xFAD male mice were bred with a C57BL/6:SJL F1 female purchased from Jackson Laboratory (Bar Harbor, ME, United States). Identification of 5xFAD progeny and WT littermates was completed by polymerase chain reaction (PCR) of transgenic human amyloid precursor protein (hAPP) from DNA tail samples. Animals were housed in a climate-controlled environment with an alternating 12 h light–dark schedule. Male and female 5xFAD and WT littermates (7.6 ± 0.6 months) were treated with or without CAW (0, 200, 500, or 1,000 mg/kg/d) in the drinking water for 5 weeks. Animals were fed PicoLab Laboratory Rodent Diet 5L0D (LabDiet, St. Louis, MO, United States). Food and water were provided ad libitum. After 5 weeks of treatment, animals were sacrificed at 9 months of age. Brains were dissected and the left cortex was used for metabolite extraction.

### 2.3 Metabolite Extraction

Methanol and water (LC-MS-grade) were purchased from EMD Millipore (Burlington, MA, United States). Formic acid (certified ACS reagent) was from Fisher Chemicals (Suwanee, GA, United States). Labeled amino acid indole-3-acetic acid (D7) was used as internal standard (Cambridge Isotope Laboratories, Inc., MA, United States). For metabolomic analysis, mice cortical tissue was extracted with a protocol previously reported with some modifications (Kirkwood et al., 2013). Briefly, the weight of one cortex from one hemisphere was measured accurately and placed in a 2 ml homogenization tube prefilled with ceramic beads (1.4 mm). A proportional amount (20 µl/mg) of homogenization solvent [methanol: water solution (8:2, v/v)] was added to each sample. Samples were spiked with the deuterated amino acid (1 µM final concentration) to account for sample degradation and homogenized using a Precellys™ 24 bead ruptor homogenizer (Bertin Technologies, MD, United States) for three consecutive cycles of 20 s at 5,000 rpm with 30 s of cooldown in between. Samples were placed at −20°C for 1 hour and centrifuged (14,000 rpm, 10 min, 4°C) to collect the supernatant. The resultant supernatant from each sample was transferred to a HPLC vial (Microsolv, Leland, NC, United States) for HPLC–HRMS/MS analysis. A quality control sample was prepared by combining 5 µl of each sample in a single vial. Prior to analysis, 3 µl of a 50 ng/ml solution of CUDA [12-[[(cyclohexylamino)carbonyl]amino]-dodecanoic acid] was added to each sample to allow for platform stability and injection volume correction.

### 2.4 HPLC-HRMS/MS Analysis

Untargeted HPLC–HRMS/MS analyses were performed using a previously published method (Kirkwood et al., 2013; Housley et al., 2018; Magana et al., 2020) with minor modifications. Briefly, data-dependent acquisition in the positive ion mode was conducted on an AB SCIEX TripleTOF^®^ 5,600 mass spectrometer (AB SCIEX, Concord, Canada) coupled to a Shimadzu Nexera HPLC system. Chromatographic separation was performed on an Inertsil Phenyl-3 column (4.6 × 150 mm, 100 Å, 5 μm; GL Sciences, Rolling Hills Estates, CA, United States) held at 50°C. A gradient with two mobile phases was used: (A) water (LC-MS grade) with 0.1% v/v formic acid; (B) methanol (LC-MS grade) with 0.1% v/v formic acid. After 1 min at 5% B, the linear elution gradient was as follows: 1 min, 5% B; 11 min, 30% B; 20 min, 100% B; 25 min, 100% B; 30 min, 5% B; and 35 min, 5% B. The injection volume was 6 μl with a flow rate of 0.4 ml/min. Samples were analyzed in a fully randomized batch. The quality control pooled sample was spiked with 5 µl of a 5-ppm solution in ethanol of the drugs verapamil and verapamil-D3 (Cambridge Isotope Laboratories, Inc., MA, United States), and analyzed every ten injections after a methanol blank sample. The isotopic pattern obtained for these internal standards was used to monitor for platform stability along the run. The IonSpray voltage was set at 4,500 V and the source temperature was 500°C. Period cycle time was 950 ms; accumulation time 100 ms; m/z scan range 100–1,400. The collision energy was set at 35 V with a collision energy spread setting of 15 V. Mass calibration of the time-of-flight (TOF) analyzer was performed automatically every five injections.

### 2.5 Metabolomics Data Processing

Raw data was loaded to PeakView™ with XIC Manager 1.2.0 (ABSciex, Framingham, MA) for peak-picking, retention time correction, and peak alignment. Metabolite identities were confirmed as previously described by matching with an *in-house* library comprising IROA standards (IROA Technology, Bolton, MA) and other commercially available standards (650 total) (García-Jaramillo et al., 2020). The list of identified peaks was exported to MultiQuant 3.0.2, and chromatograms integrated to obtain peak area values for all the assigned metabolites. To account for drift during the metabolomics run, and for potential differences in the injection volume, the annotated metabolites were normalized to the highest peak area measured for the internal standard CUDA. A complete list of annotated metabolites is provided in Supplementary Table S1.

### 2.6 Statistical Analysis

To compare the cortical metabolomic profiles of untreated WT and 5xFAD transgenic mice, peak intensities of individual metabolites were log_2_-transformed and mean-normalized by dividing each value by the within-mouse average (across all metabolites) and then scaling by the average of all values (across all metabolites and mice). Heatmaps of peak intensities across all metabolites were created to descriptively assess for differences in magnitude by sex and genotype. To identify changes in the metabolomic profiles, a linear regression model for the normalized log_2_ intensities was implemented with indicator variables for sex, genotype, and the interaction between sex and genotype. Within-sex and within-genotype fold changes were back-transformed and reported, as well as the marginal effects for sex and genotype. For all comparisons, volcano plots were created with a horizontal line to indicate the *p*-value cutoff for the Benjamini-Hochberg control of false discovery rate, which was computed using an overall significance level of 0.05.

The same normalization and descriptive analysis described above were used for the CAW dose-response analysis. For each sex and genotype combination, the normalized log_2_ intensities were plotted by dose then separate one-way ANOVAs were conducted with dose as the independent variable for each metabolite. The overall Benjamini-Hochberg adjusted *p*-values from the ANOVAs were reported. Pairwise t-tests for all possible comparisons were conducted with a Benjamini-Hochberg *p*-value adjustment. In addition, separate 3-way ANOVAs were conducted on the normalized log_2_ intensities for each metabolite to determine if a significant dose-response curve exists and whether it differs by sex and genotype. The least squares slopes for the overall dose-response, the marginal dose-response by sex and genotype, and the within-sex-and-genotype dose-response were computed and reported. The fold changes from a dose of 0–1,000 were calculated using the slopes, and volcano plots of the fold changes with the Benjamini-Hochberg FDR *p*-value cutoff were generated. All analyses were conducted in StataSE 16.1 and R version 4.0.4. Partial least squares-discriminant analysis and plots (PLS-DA) were generated with MetaboAnalyst 5.0 (Pang et al., 2021).

### 2.7 Metabolomic Pathway Analysis

Metabolite data was imported into MetaboAnalyst 5.0 (Pang et al., 2021) for pathway analysis to compare the metabolomic profiles of WT and 5xFAD mice and to investigate the effects of 500 mg/kg/d CAW on metabolomic profiles in WT and 5xFAD mice. A suitable match for the metabolite maleimide could not be found in MetaboAnalyst and so 119 of 120 annotated metabolites were included in the pathway analyses. A threshold of 0.1 was set for the impact value and a raw *p* value ≤0.05 was considered significant.

## 3 Results

### 3.1 Cortical Metabolomic Profiles of Untreated WT and 5xFAD Transgenic Mice

**FIGURE 1:**
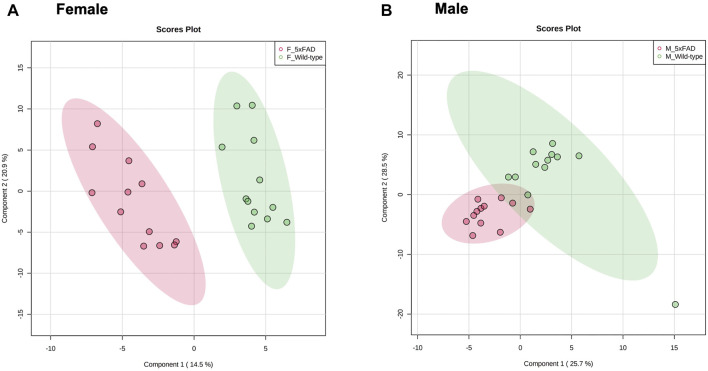
PLS-DA plots of untreated female and male 5xFAD mice compared to untreated wild-type (WT) mice.

Cortical tissue from untreated (0 mg/kg/d CAW) male and female WT and 5xFAD mice was collected and a PLS-DA analysis was performed using 120 metabolites (Supplementary Table S1) identified through methods described in Section 2.5. As seen in Figure 1, there was separation between the WT and 5xFAD genotypes in both female (1A) and male (1B) mice, though the separation was more clearly defined in female mice than in male mice.

**FIGURE 2:**
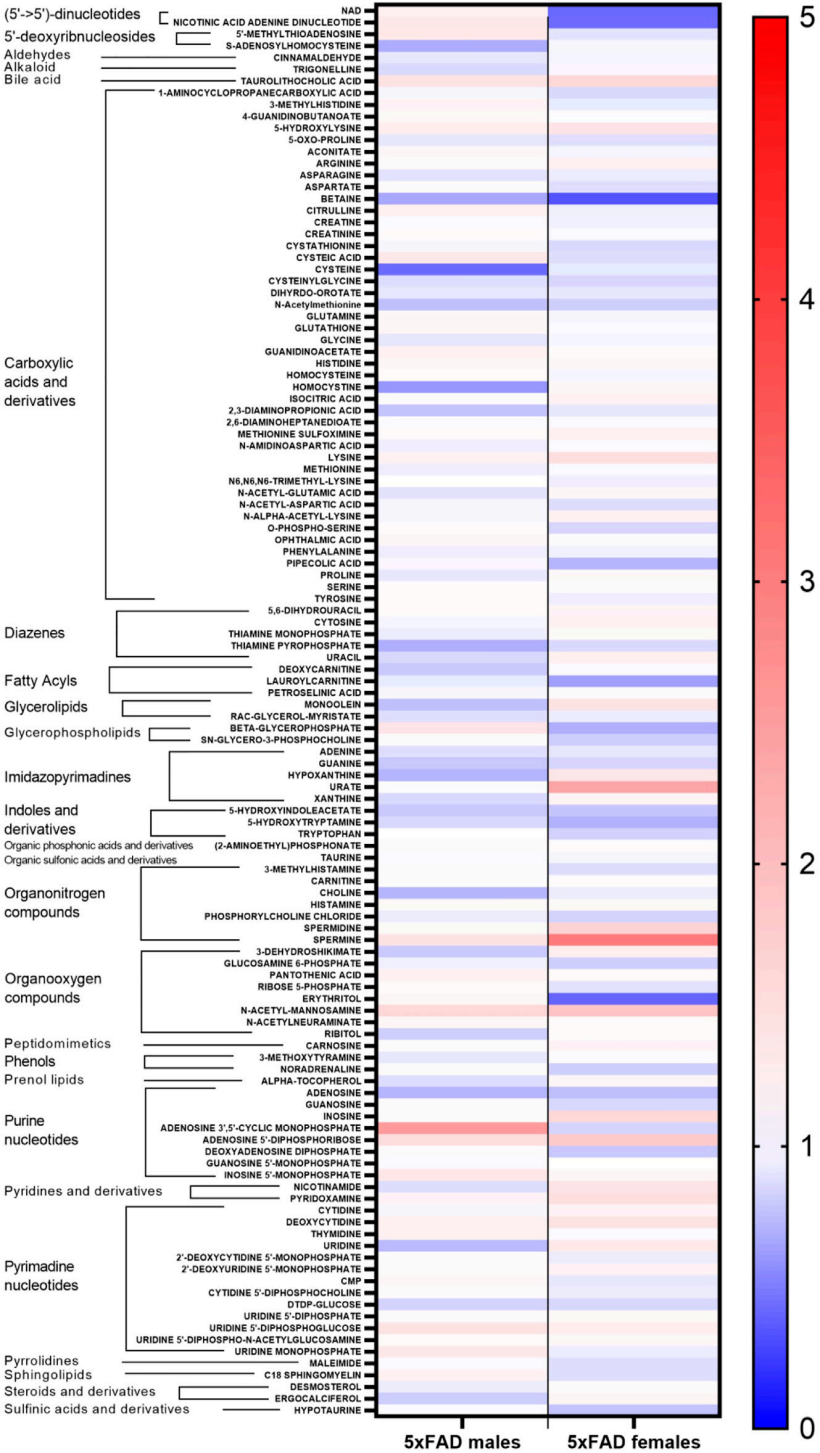
Heatmap of changes in identified metabolites in male and female 5xFAD mice compared to male and female wild-type (WT) mice, respectively.

Statistically significant fold changes for metabolites were determined for each sex using a full factorial per metabolite model with a linear regression model estimator and Benjamini and Hochberg false discovery rate corrections (Supplementary Table S2). Relative changes in individual metabolites for male and female 5xFAD mice compared to WT littermates of the same sex are shown in Figure 2. While there were similarities between male and female mice in the direction of change for most individual metabolites resulting from transgenic status, there were clearly observable sex-related differences in either the magnitude of change or the direction of change for several metabolites.

**FIGURE 3:**
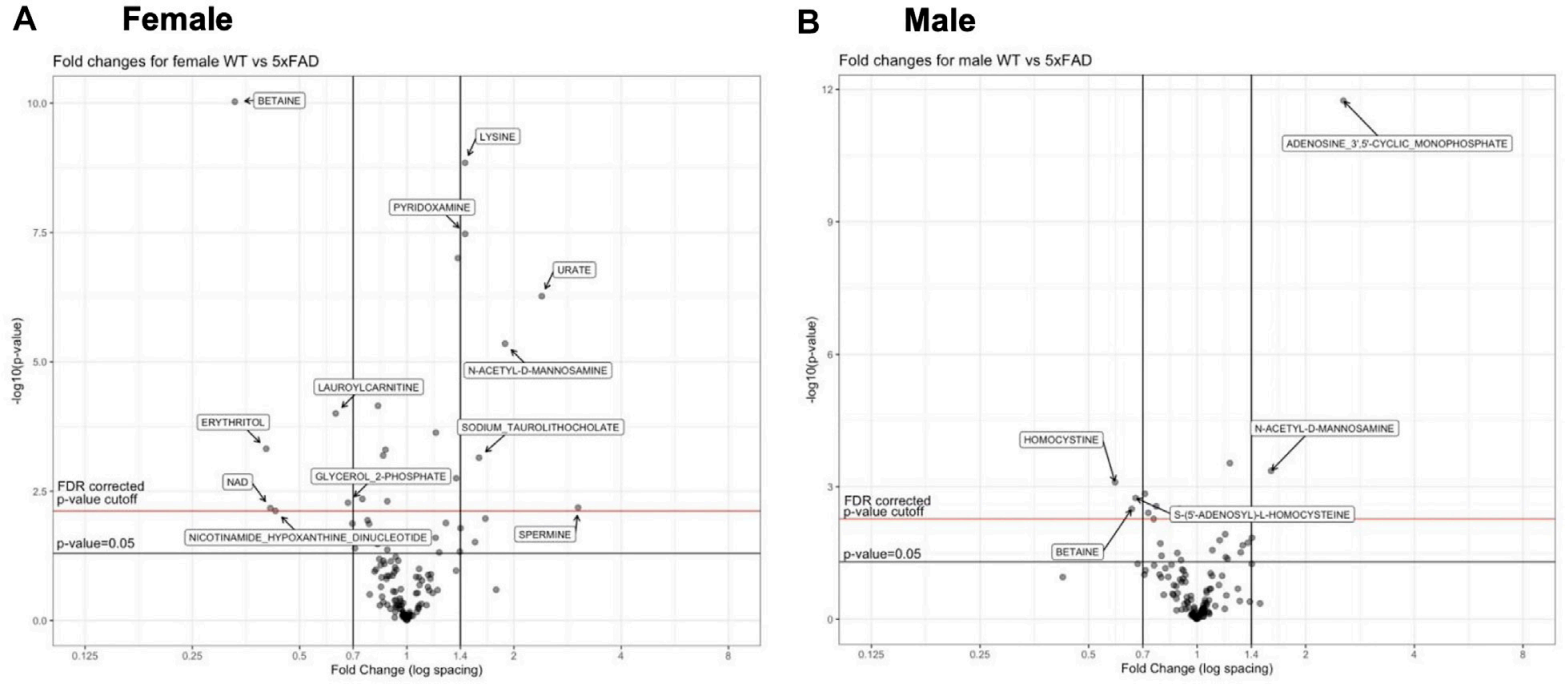
Volcano plots of metabolities that changed significantly in 5xFAD mice as compared to wild-type (WT) mice.

Individual metabolites that were significantly altered in 5xFAD mice compared to WT mice can be seen in the volcano plots for female (Figure 3A) and male mice (Figure 3B). The experimental resolution used in the statistical analysis was such that fold changes between 0.7 and 1.4 could not be distinguished from measurement nonlinearities. Therefore, on the volcano plots, only metabolites outside of those thresholds (indicated by vertical lines) and above the indicated FDR cutoff (red horizontal line) can be considered statistically significant. Overall, 12 metabolites (6 upregulated and 6 downregulated) were significantly altered by the transgene in female 5xFAD mice, whereas there were five significantly altered metabolites (2 upregulated and 3 downregulated) in male 5xFAD (Figure 3). Of these, betaine and N-acetyl-D-mannosamine were the only metabolites that were significantly altered in both male and female 5xFAD mice compared to their sex-matched WT littermates.

A metabolomic pathway analysis was performed using MetaboAnalyst 5.0 to further compare WT and 5xFAD transgenic mice. Results are presented in Table 2. The total number of significantly altered pathways with an impact score ≥0.1 was eleven in 5xFAD females and two in 5xFAD males (Table 2). Amino sugar and nucleotide sugar metabolism was the only metabolic pathway with an impact score ≥0.1 that was significantly altered in both 5xFAD females (*p* < 0.001) and 5xFAD males (*p* = 0.027). Significantly altered pathways with the highest impact scores in 5xFAD females included nicotinate and nicotinamide metabolism (impact score = 0.639, *p* = 0.005), glycine, serine, and threonine metabolism (impact score = 0.591, *p* = 0.006), and pyrimidine metabolism (impact score = 0.493, *p* = 0.016). Pentose phosphate and glucuronate interconversion was also significantly impacted in 5xFAD males (impact score 0.250, *p* = 0.03). Taken together, the results of metabolomic analyses comparing untreated WT and 5xFAD mice indicate that there are significant metabolic alterations associated with the 5xFAD transgene but there is variability in the number, magnitude, and direction of these changes between female and male mice.

**TABLE 2:** Pathway analysis comparing male and female 5xFAD mice to male and female wild-type (WT) mice, respectively.

Metabolic pathway	Compounds	Hits	Raw *p* value		Impact score
			Male 5xFAD	Female 5xFAD	
Nicotinate and nicotinamide metabolism	15	4	0.467	0.005	0.639
Glycine, serine and threonine metabolism	34	9	0.349	0.006	0.591
Pyrimidine metabolism	39	13	0.170	0.016	0.493
Arginine biosynthesis	14	6	0.358	0.017	0.365
Arginine and proline metabolism	38	8	0.430	0.024	0.316
Purine metabolism	66	15	0.078	0.012	0.302
Tryptophan metabolism	41	3	0.648	0.026	0.262
Pentose and glucuronate interconversions	18	2	0.030	0.240	0.250
Amino sugar and nucleotide sugar metabolism	37	4	0.030	<0.001	0.186
beta-Alanine metabolism	21	7	0.237	<0.001	0.168
Aminoacyl-tRNA biosynthesis	48	14	0.224	0.026	0.167
Glycerophospholipid metabolism	36	4	0.361	0.028	0.103
Vitamin B6 metabolism	9	1	0.027	<0.001	0.078
Pantothenate and CoA biosynthesis	19	5	0.204	0.002	0.029
Lysine degradation	25	5	0.046	<0.001	0.005
Galactose metabolism	27	1	0.029	0.231	0.002
Biotin metabolism	10	1	0.015	<0.001	0
Ether lipid metabolism	20	1	0.419	0.013	0
Ascorbate and aldarate metabolism	10	1	0.029	0.231	0

### 3.2 Dose-Response Effects of CAW on Cortical Metabolomic Profiles

**FIGURE 4:**
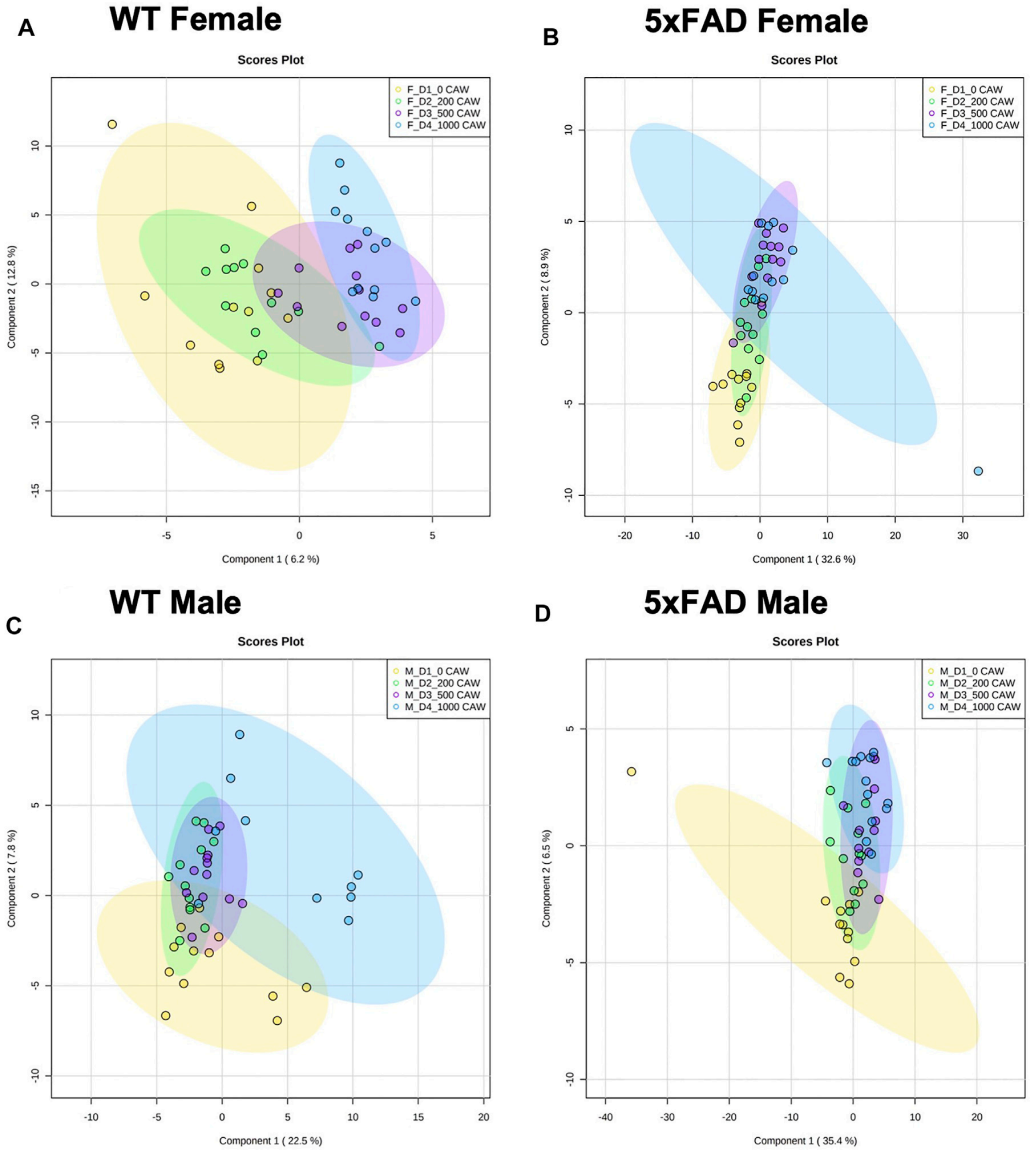
PLS-DA plot comparing cortical metabolomic profiles of male and female 5xFAD and wild-type (WT) mice treated with CAW 0 (D1), 200 (D2), 500 (D3), or 1,000 (D4) mg/kg.

**FIGURE 5:**
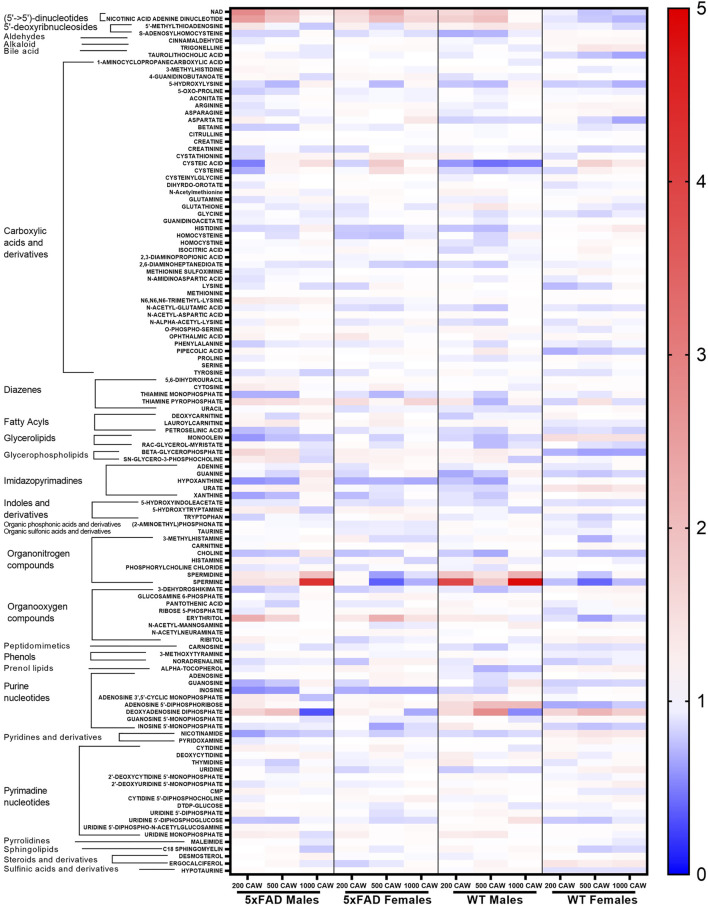
Heatmap of changes in identified metabolites in the cortex of male and female 5xFAD and wild-type (WT) mice treated with CAW (200, 500, and or 1,000 mg/kg) compared to untreated animals of the same genotype and gender.

Male and female WT and 5xFAD mice were treated with CAW in their drinking water for 5 weeks at four different doses (0, 200, 500, and 1,000 mg/kg/d) and changes in cortical metabolomic profiles were assessed. A PLS-DA analysis was performed for each group using the same 120 identified metabolites (Supplementary Table S1). The PLS-DA plots for WT females (Figure 4A), 5xFAD females (Figure 4B), WT males (Figure 4C) and 5xFAD males (Figure 4D) showed separation between control and CAW-treated animals, particularly at the higher CAW concentrations.

**FIGURE 6:**
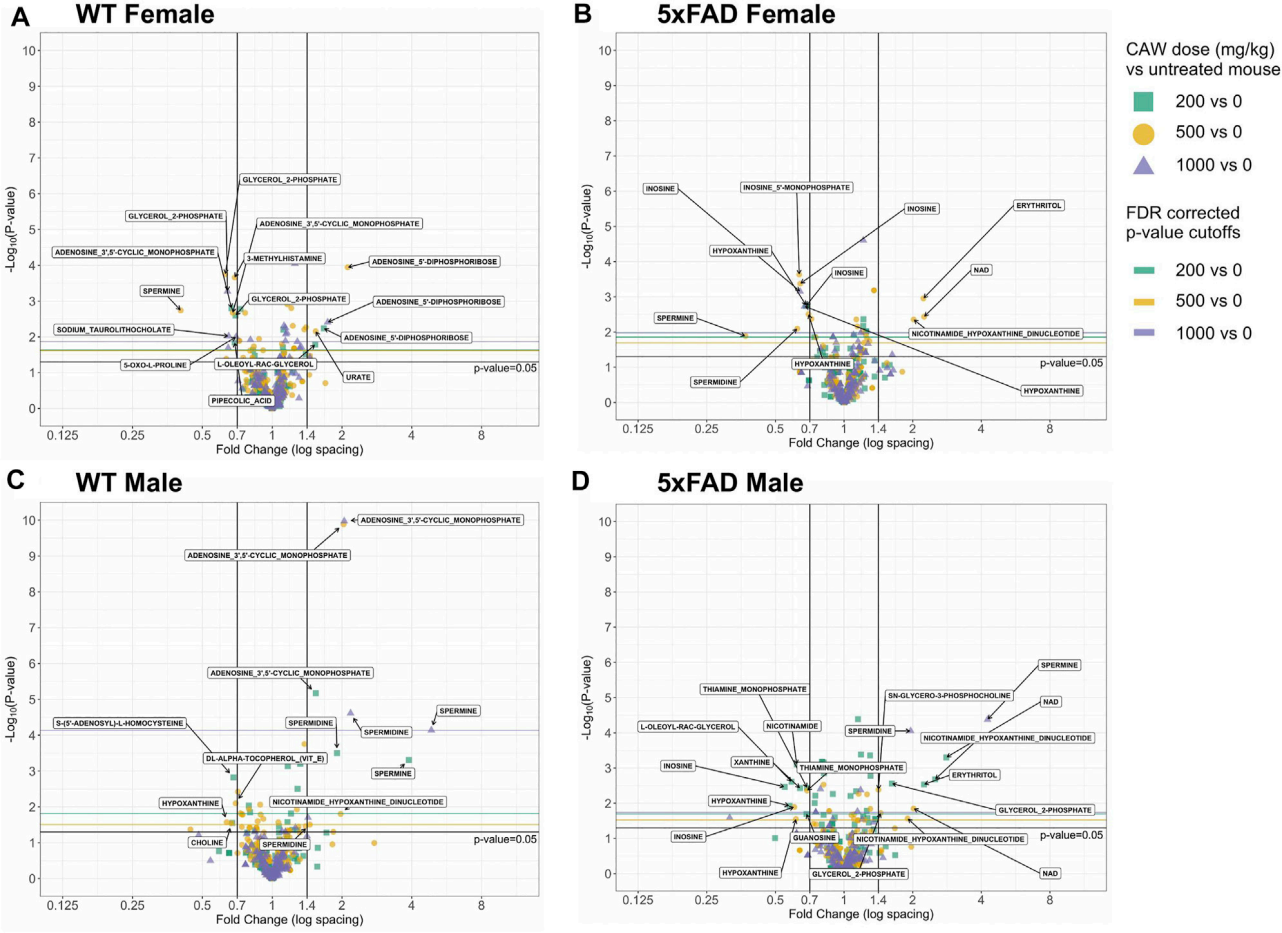
Volcano plots of cortical metabolites female and male wild-type (WT) and 5xFAD mice treated with CAW (200, 500 or 1,000 mg/kg) compared to untreated sex- and genotype-matched mice.

A heat map of the dose-response effects of CAW on the cortical metabolomic profiles of male and female WT and 5xFAD mice compared to genotype- and sex-matched control animals is shown in Figure 5. While there were some similarities between sexes and/or genotypes in response to different concentrations of CAW, changes in individual metabolites were rarely consistent across all four groups of animals (WT females, 5xFAD females, WT males, 5xFAD males). Fold changes were calculated, and statistical significance determined using a linear regression model for each metabolite with log_2_ peak intensity as the outcome and treatment dose as the independent variable. Pairwise t-tests for the normalized metabolite intensities at each concentration of CAW compared to genotype- and sex-matched control animals were conducted using a Benjamini Hochberg FDR correction (Benjamini and Hochberg, 1995) (Figure 6). Again, significantly altered metabolites were those with fold changes outside of the 0.7–1.4 range with *p* values lower than the FDR correction limit when compared to genotype- and sex-matched control animals (Figures 6A–D).

Metabolite changes varied in response to different doses of CAW and there were only a few examples of individual metabolites that were significantly altered at all three doses of CAW for any given genotype. In WT females, glycerol 2-phosphate was significantly downregulated and adenosine 5′-diphosphoribose was significantly upregulated at all three dose levels (Figure 6A). In 5xFAD females, inosine and hypoxanthine were significantly downregulated at all three dose levels (Figure 6B). In WT males, adenosine 3′,5′-cyclic monophosphate and spermidine were significantly upregulated at all three dose levels (Figure 6C). For 5xFAD males, there were several metabolites that were significantly altered at each dose level, but no single metabolite was significantly altered at all three dose levels (Figure 6D). Across all genotypes and sexes (WT females, 5xFAD females, WT males, 5xFAD males), we observed that there were more significantly changed metabolites at the 500 mg/kg/d CAW dose level (24 metabolites total) than at the 200 mg/kg/d (20 metabolites) or 1,000 mg/kg/d (9 metabolites) dose levels. In addition, there were four examples of metabolites that were significantly altered in 5xFAD mice compared to WT mice which were then significantly reversed by CAW treatment (erythritol, nicotinamide adenine dinucleotide (NAD+), nicotinamide hypoxanthine dinucleotide, spermine); these changes all occurred only in 5xFAD females and only at the 500 mg/kg/d dose level (Figures 3A, 6B).

Based on these results, we conducted a metabolomic pathway analysis for female and male 5xFAD and WT mice treated with 500 mg/kg/d CAW compared to genotype- and sex-matched control mice. All metabolic pathways that were significantly altered (raw *p* value <0.05) in at least one of the four groups are presented in Table 3 with their corresponding impact scores. Nine pathways were significantly altered in 5xFAD females, followed by seven pathways in 5xFAD males, six pathways in WT females, and four pathways in WT males. Three pathways with an impact score ≥0.1 were found to be significantly altered in at least three of the four groups (WT females, WT males, 5xFAD females, 5xFAD males): purine metabolism, nicotinate and nicotinamide metabolism, and glycerophospholipid metabolism.

**TABLE 3:** Pathway analysis in mice treated with CAW (500 mg/kg) vs. sex- and genotype-matched untreated controls.

Metabolic pathway	Compounds	Hits	Raw *p* value				Impact score
			WT Female	5xFAD Female	WT Male	5xFAD Male	
Taurine and hypotaurine metabolism	8	4	0.520	0.039	0.063	0.925	0.714
Thiamine metabolism	7	3	0.686	0.019	0.282	0.133	0.667
Nicotinate and nicotinamide metabolism	15	4	0.098	0.010	0.027	0.007	0.639
Pyrimidine metabolism	39	13	0.227	0.131	0.131	0.015	0.493
Glutathione metabolism	28	8	0.089	0.047	0.021	0.352	0.419
Arginine biosynthesis	14	6	0.483	0.342	0.342	0.038	0.365
Arginine and proline metabolism	38	8	0.141	0.130	0.049	0.507	0.316
Purine metabolism	66	15	0.011	0.003	0.044	0.047	0.302
beta-Alanine metabolism	21	7	0.024	0.146	0.086	0.194	0.168
Aminoacyl-tRNA biosynthesis	48	14	0.041	0.659	0.490	0.695	0.167
Glyoxylate and dicarboxylate metabolism	32	4	0.306	0.018	0.436	0.683	0.148
Glycerophospholipid metabolism	36	4	0.050	0.012	0.099	0.033	0.103
Citrate cycle (TCA cycle)	20	2	0.994	0.008	0.533	0.552	0.102
Vitamin B6 metabolism	9	1	0.007	0.510	0.243	0.745	0.078
Panthothenate and CoA biosynthesis	19	5	0.290	0.323	0.224	0.004	0.029
Ether lipid metabolism	20	1	0.217	0.022	0.080	0.003	0
Biotin metabolism	10	1	0.008	0.987	0.173	0.608	0

**FIGURE 7:**
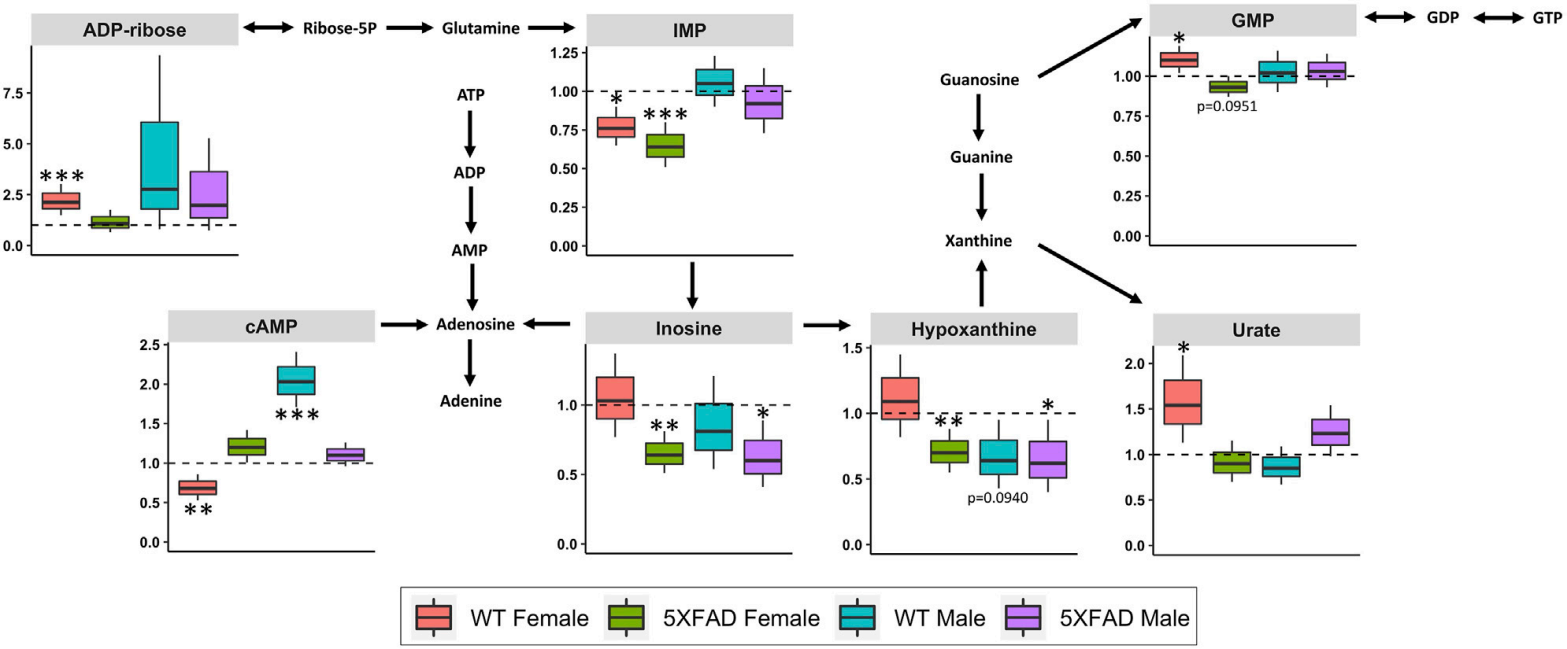
Fold changes in cortical metabolites related to purine metabolism in male and female 5xFAD or wild-type (WT) mice following treatment with CAW (500 mg/kg) compared to sex- and genotype-matched untreated controls. *p **<** 0.05, **p **<** 0.005, ***p **<** 0.001.

Purine metabolism (impact score = 0.302; Table 3) was significantly altered by CAW treatment (500 mg/kg/d) in all four groups: 5xFAD females (*p* = 0.003), WT females (*p* = 0.011), 5xFAD male (*p* = 0.047) and WT males (*p* = 0.044). Fifteen of the 120 annotated metabolites in the dataset were involved in the purine metabolism pathway. Figure 7 shows the purine metabolism pathway with box-and-whisker plots for individual metabolites that significantly changed in at least one of the four groups. Inosine and hypoxanthine were significantly decreased by CAW treatment in both 5xFAD male (*p* = 0.026, *p* = 0.042, respectively) and female (*p* = 0.002, *p* = 0.006, respectively) mice. Three metabolites (guanosine 5′-monophosphate (GMP), urate, adenosine diphosphate (ADP)-ribose) were significantly increased in WT females only (*p* = 0.031, *p* = 0.042, and *p* = 0.001, respectively). Inosine 5′-monophosphate (IMP) was significantly decreased in both 5xFAD females (*p* = 0.001) and WT females (*p* = 0.013). Cyclic adenosine monophosphate (cAMP) was significantly increased in WT males (*p* < 0.001) and significantly decreased in WT females (*p* = 0.006).

**FIGURE 8:**
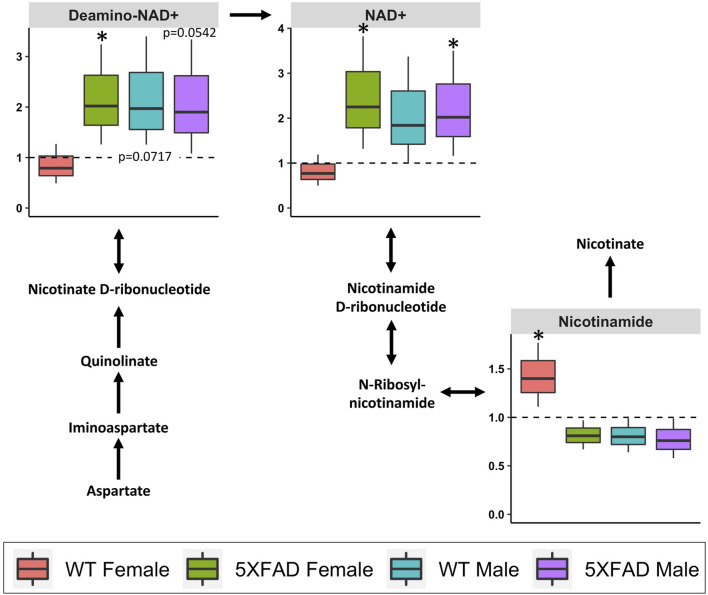
Fold changes in cortical metabolites related to nicotinate and nicotinamide metabolism in male and female 5xFAD or wild-type (WT) mice following treatment with CAW (500 mg/kg) compared to sex- and genotype-matched untreated controls. *p **<** 0.05, **p **<** 0.005, ***p **<** 0.001.

Nicotinate and nicotinamide metabolism (impact score = 0.639; Table 3) was significantly altered by CAW treatment (500 mg/kg/d) in 5xFAD females (*p* = 0.010), 5xFAD males (*p* = 0.007) and WT males (*p* = 0.007), but not in WT females (*p* = 0.098). Of the 120 identified metabolites used in the analysis, four metabolites (NAD+, deamino-NAD+, nicotinamide, and aspartate) were involved in the nicotinate and nicotinamide metabolism pathway. As before, box-and-whisker plots in Figure 8 show metabolites that were significantly changed in at least one group. NAD+ was significantly increased in both 5xFAD males (*p* = 0.028) and 5xFAD females (*p* = 0.022). In addition, deamino-NAD+ was significantly increased in 5xFAD females (*p* = 0.026) and nicotinamide was significantly increased in WT females (*p* = 0.032).

**FIGURE 9:**
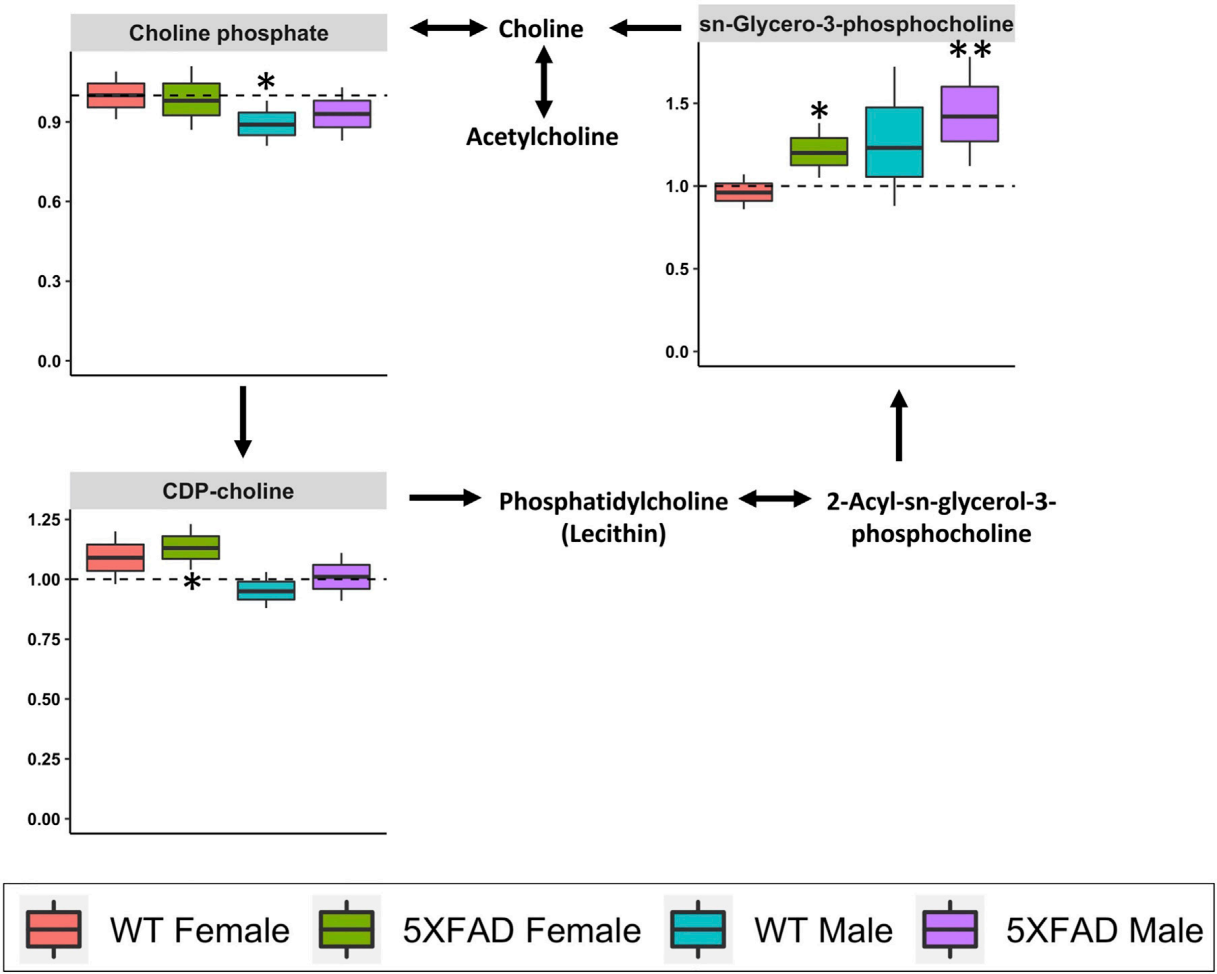
Fold changes in cortical metabolites related to glycerophospholipid metabolism in male and female 5xFAD or wild-type (WT) mice following treatment with CAW (500 mg/kg) compared to sex- and genotype-matched untreated controls. *p **<** 0.05, **p **<** 0.005, ***p **<** 0.001.

Glycerophospholipid metabolism (impact score = 0.103; Table 3) was significantly altered by CAW treatment (500 mg/kg/d) in WT females (*p* = 0.050), 5xFAD females (*p* = 0.012) and 5xFAD males (*p* = 0.033), but not WT males (*p* = 0.099). Four of the 120 identified metabolites in our study (choline, cytidine 5′-diphospho (CDP)-choline, choline phosphate, and sn-glycero-3-phosphocholine) were involved in glycerophospholipid metabolism. Box-and-whisker plots in Figure 9 show metabolites involved in glycerophospholipid metabolism that were significantly changed in at least one of the four groups. The metabolite sn-Glycero-3-phosphocholine was significantly increased in 5xFAD males (*p* = 0.008) and 5xFAD females (*p* = 0.029), while CDP-choline was significantly increased in 5xFAD females only (*p* = 0.013). Choline phosphate was significantly decreased in WT males (*p* = 0.041).

## 4 Discussion

Our group has previously reported that eight-month-old male and female 5xFAD mice and WT littermates treated with CAW (200, 500, or 1,000 mg/kg/d) in their drinking water for 5 weeks displayed a dose-dependent improvement in memory in both sexes and genotypes and without altering Aβ plaque burden in the 5xFAD mice (Matthews et al., 2019). To further investigate potential mechanisms underlying these cognitive-enhancing effects of CAW, a metabolomic analysis was performed on cortical samples collected from the 5xFAD and WT mice used in that prior study.

Cortical metabolomic profiles of untreated 5xFAD mice and WT littermates showed variations between male and female mice in the metabolites altered by the transgenic status (Figures 2, 3), with a greater number of metabolites being significantly altered in females. This finding may be related to other sex differences observed in the 5xFAD transgene model of AD. Studies in 5xFAD mice have found that female mice display greater molecular pathology (Sadleir et al., 2015; Bundy et al., 2019), higher levels of cerebral Aβ42 (Sadleir et al., 2015), and a greater Aβ plaque burden than male mice (Bhattacharya et al., 2014; Reid and Darvesh, 2015). Similar sex-related differences in pathology have also been observed in other transgenic models of AD, including the APP/PS1 (Li et al., 2016; Mifflin et al., 2021) and 3xTg-AD (Carroll et al., 2010; Yang et al., 2018) models.

A metabolomic pathway analysis comparing untreated male and female 5xFAD mice to WT littermates similarly found differences between sexes. In total, eleven metabolic pathways with an impact score ≥0.1 were significantly altered in 5xFAD females compared to WT females while only two pathways were significantly altered in 5xFAD males compared to WT males. Notably**,** several of the pathways altered in 5xFAD females, including purine metabolism, pyrimidine metabolism, glycerophospholipid metabolism, glycine, serine and threonine metabolism, arginine and proline metabolism, are in agreement with findings from previous metabolomics studies in other mouse models of Aβ accumulation (González-Domínguez et al., 2014; González-Domínguez et al., 2015; Dejakaisaya et al., 2021; Zhao et al., 2021).

Next, the effects of three concentrations of CAW (200, 500 and 1,000 mg/kg/d) on cortical metabolomic profiles of male and female 5xFAD mice and their WT littermates were examined. As seen in Figures 5, 6, there was substantial variation between sexes and genotypes in the direction and magnitude of change of individual metabolites in response to CAW, with very few metabolites being significantly altered at all three concentrations of CAW.

A pathway analysis was conducted in male and female 5xFAD and WT mice treated with 500 mg/kg/d CAW compared to sex- and genotype-matched controls. This dose of CAW was chosen because it elicited the greatest total number of significantly altered metabolites overall compared to the other two dose levels. The 500 mg/kg/d CAW dose also reversed more of the significant metabolite changes observed in untreated 5xFAD mice compared to WT mice than did the other CAW doses. A pathway analysis identified three pathways (purine metabolism, nicotinate and nicotinamide metabolism, and glycerophospholipid metabolism) that were all significantly altered by CAW in at least three of the four groups: WT female, 5xFAD female, WT male, 5xFAD male (Table 3). Evidence in the literature suggests that these three pathways may be involved in the pathophysiology of AD and therefore, these findings may provide new insight into the mechanisms underlying the cognitive-enhancing effects of CAW (Gray et al., 2018a; Matthews et al., 2019; Zweig et al., 2021).

Purine metabolism was significantly altered by CAW (500 mg/kg/d) in WT males, 5xFAD males, WT females, and 5xFAD females (Table 3). The magnitude and direction of change for individual metabolites involved in purine metabolism varied by treatment group (Figure 7). It is notable that purine metabolism was significantly altered in untreated 5xFAD females compared to WT females in our study, and that dysregulation of purine metabolism has previously been demonstrated in APP/PS1 (González-Domínguez et al., 2014; González-Domínguez et al., 2015) and 3xTgAD (Esteve et al., 2017; Zhao et al., 2021) mouse models of AD as well. These data may have clinical significance since evidence of dysregulated purine metabolism has been seen in the cerebrospinal fluid (Kaddurah-Daouk et al., 2013) and post-mortem brain samples collected from patients with AD (Ansoleaga et al., 2015; Alonso-Andrés et al., 2018; Mahajan et al., 2020), with evidence of disease stage- and brain region-dependent metabolite changes.

The most notable changes in individual metabolites involved in purine metabolism following CAW treatment were inosine and hypoxanthine, both of which were significantly decreased in 5xFAD males and 5xFAD females. Inosine, a purine nucleoside produced through the catabolism of adenosine (Teixeira et al., 2020), is increased in various animal models of AD (González-Domínguez et al., 2014; González-Domínguez et al., 2015; Esteve et al., 2017) and in several brain regions in post-mortem brain samples from patients with AD. (Alonso-Andrés et al., 2018) This suggests that CAW may act by normalizing inosine levels, though inosine was not elevated in untreated male and female 5xFAD mice in this study. Interestingly, there is also evidence that treatment with inosine can improve cognitive deficits in rodent models of aging and AD. (Ruhal and Dhingra, 2018; Teixeira et al., 2020) In aged female rats, inosine significantly improved cognitive function, while also showing antioxidant and anti-inflammatory effects (Ruhal and Dhingra, 2018). Similarly, in a rat model of streptozotocin (STZ)-induced AD, inosine attenuated STZ-induced memory impairments, reduced acetylcholinesterase activity, prevented alterations in ion pump activities, and demonstrated antioxidant activity (Teixeira et al., 2020). This apparent discrepancy may potentially reflect an issue of inosine utilization. The elevated levels previously reported in patients with AD and transgenic mouse models of AD could suggest poor utilization of inosine; our findings may therefore indicate that CAW improves inosine utilization, resulting in lower inosine levels, but accompanied by improved cognition.

Hypoxanthine is a purine derivative formed as a result of DNA metabolism after apoptosis and cell lysis (Chouraki et al., 2017). While there is evidence to suggest that hypoxanthine is dysregulated in animal models of AD, the evidence is inconsistent, which may reflect disease stage-, species-, and/or brain region-specific changes (Esteve et al., 2017; Zhao et al., 2021). The evidence from human studies is inconclusive as well. One study found that hypoxanthine was significantly decreased in the frontal cortex of patients with AD compared to healthy controls, with no significant changes in the parietal or temporal cortices (Alonso-Andrés et al., 2018). Conversely, a metabolomics study of cerebrospinal fluid samples from patients with AD and mild cognitive impairment (MCI) found that hypoxanthine levels were unchanged in AD, but were significantly increased in MCI compared to controls (Kaddurah-Daouk et al., 2013). While there is ample evidence to suggest that purine metabolism is dysregulated in AD, it is difficult to attribute the changes in specific purine metabolites (e.g., inosine and hypoxanthine) observed in this study to the cognitive-enhancing effects of CAW without further targeted experiments.

Treatment with CAW (500 mg/kg/d) significantly altered nicotinate and nicotinamide metabolism in 5xFAD males, 5xFAD females, and WT males, but not WT females (Table 3). The most interesting changes were seen for the metabolite NAD+, an important coenzyme in the body that is involved in a variety of reactions, including as part of several key metabolic pathways (e.g., glycolysis, the citric acid cycle, and oxidative phosphorylation) (Xing et al., 2019). Studies in humans, rodents, and other organisms have shown that NAD+ levels decline in normal aging (Lautrup et al., 2019), and NAD+ depletion and disruption of NAD+-involved pathways have been implicated in the pathophysiology of AD. (Lautrup et al., 2019)

Dysregulated nicotinate and nicotinamide metabolism has been observed in 5xFAD (Kim et al., 2020), PS1 (Trushina et al., 2012), and 3xTgAD (Zhao et al., 2021) mouse models of AD, although the specific metabolites as well as magnitude and direction of changes varied depending on the age and genotype. In the present study, nicotinate and nicotinamide metabolism was significantly altered in untreated 5xFAD females compared to WT females. Altered NAD+ levels have also been observed in fibroblasts collected from patients with late-onset AD. (Sonntag et al., 2017) Therapeutic interventions that target the nicotinate and nicotinamide pathways have likewise shown potential as cognitive-enhancing agents. NAD+ (Xing et al., 2019) and its precursors (nicotinamide (Green et al., 2008; Liu et al., 2013), nicotinamide ribose (Gong et al., 2013; Hou et al., 2018; Xie et al., 2019), and nicotinamide mononucleotide (Wang et al., 2016)) have all been found to improve cognitive performance in a variety of AD mouse models.

The implication of the nicotinate and nicotinamide pathway for the mechanism of action of CAW is in line with our previous work showing that CAW improves mitochondrial function, reduces oxidative stress, and increases synaptic density (Gray et al., 2015; Gray et al., 2017b; Gray et al., 2018a; Matthews et al., 2019). These effects are similar to what has been observed in mouse models following treatment with NAD+ or its precursors, in which treatment was also associated with cognitive enhancement (Liu et al., 2013; Long et al., 2015; Wang et al., 2016; Hou et al., 2018; Kim et al., 2020). Therefore, the observed upregulation of cortical levels of NAD+ by CAW could be mediating the effects of CAW on mitochondrial function, oxidative stress, and synaptic density, and thereby contributing to its cognitive-enhancing effects.

Glycerophospholipid metabolism was also significantly affected by treatment with CAW 500 mg/kg/d in 5xFAD females, 5xFAD males, and WT females, but not WT males (Table 3). Dysregulation of glycerophospholipid metabolism in AD has previously been demonstrated in post-mortem brain samples from AD patients (Nitsch et al., 1991) and in 2- and 6-month-old 3xTgAD mice (Zhao et al., 2021). Glycerophospholipid metabolism was also significantly altered in untreated 5xFAD females compared to WT females in the present study. Much of the research into glycerophospholipid metabolism in AD has focused on choline-containing phospholipids based on the observed decline in cholinergic transmission present in AD. (Tayebati and Amenta, 2013) Choline is an essential nutrient, a precursor of acetylcholine (ACh), and a necessary component for many membrane phospholipids, and it is hypothesized that the breakdown of membrane phospholipids may play a role in the pathogenesis of AD. (Tayebati and Amenta, 2013)

One potentially important finding from our study was that CAW significantly increased cortical levels of the choline-containing compound sn-Glycero-3-phosphocholine in both male and female 5xFAD mice. Also known as glycerophosphocholine (GPC) or choline alfoscerate, sn-Glycero-3-phosphocholine is a derivative of phosphatidylcholine and an ACh precursor (De Jesus Moreno Moreno, 2003). In both *in vitro* and *in vivo* studies, GPC has shown neuroprotective and cognitive-enhancing activities via effects on levels of brain-derived neurotrophic factor (BDNF) (Catanesi et al., 2020) and choline acetyltransferase (Lee et al., 2017), the enzyme responsible for ACh synthesis. Promising effects of GPC treatment on cognition (De Jesus Moreno Moreno, 2003) and dementia-related behavior (Carotenuto et al., 2017) have also been observed in patients with mild to moderate AD.

In addition to the observed effects on GPC, CAW also significantly increased the choline-containing metabolite CDP-choline (also known as citicoline) in the cortex of 5xFAD females. Similar to GPC, citicoline has demonstrated considerable potential as an adjunct therapy for AD in pre-clinical and clinical studies (Secades, 2019; Piamonte et al., 2020).

Based on the observed effects of CAW on glycerophospholipid metabolism, one potential novel mechanism of action underlying CAW’s cognitive benefits may be BDNF modulation. Indeed, various *Centella asiatica* extracts have been shown to upregulate BDNF expression in animal studies (Ar Rochmah et al., 2019; Sari et al., 2019; Sbrini et al., 2020). Observational studies have demonstrated that patients with AD have lower serum levels of BDNF than healthy controls (Ng et al., 2019) and it has been hypothesized that this deficiency could play a role in the onset of AD neurodegeneration (Giuffrida et al., 2018). BDNF is currently being studied as a potential biomarker for early detection of AD (Mori et al., 2021) and is being used as a gene therapy in a phase I trial of patients with AD. (LaFee, 2021)

In conclusion, we found that the improvements in memory seen in WT and 5xFAD mice treated with CAW in our previous study were associated with modulation of several key metabolic pathways known to be dysregulated in Alzheimer’s disease: purine metabolism, nicotinate and nicotinamide metabolism, and glycerophospholipid metabolism. Modulation of these pathways and the individual metabolites associated with these pathways may represent novel mechanisms of action underlying the cognitive-enhancing effects of CAW and may be related to other targeted mechanisms we have previously reported in our work with CAW (Gray et al., 2015; Gray et al., 2016; Gray et al., 2018a; Gray et al., 2018b; Matthews et al., 2019; Zweig et al., 2021). For each of these pathways, future studies analyzing gene or protein expression of enzymes involved in these pathways and/or co-treating with specific inhibitors are needed to confirm their involvement in the cognitive enhancing mechanism of CAW. Furthermore, modulation of these pathways by CAW in humans will need to be confirmed potentially through biomarker analysis of plasma or cerebrospinal fluid samples from clinical trial participants.

## Data Availability

The raw data supporting the conclusion of this article will be made available by the authors, without undue reservation.
