# Supplementary figures and images for: Centella asiatica Alters Metabolic Pathways Associated With Alzheimer’s Disease in the 5xFAD Mouse Model of ß-Amyloid Accumulation
Source: Front Pharmacol. 2021 Dec 16;12:788312. doi: 10.3389/fphar.2021.788312 (PMC8717922; doi:10.3389/fphar.2021.788312)

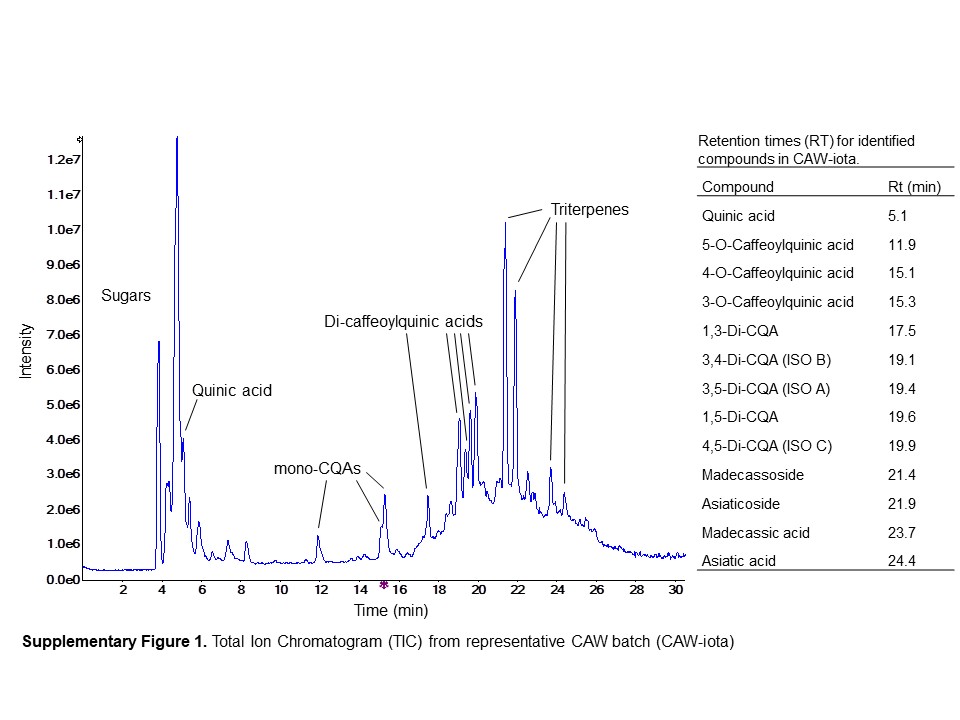

Supplement: Supplementary file 1 [file Image1.JPEG]
